# Surgical Strategies and Results for Repair of Pulmonary Atresia with Ventricular Septal Defect and Major Aortopulmonary Collaterals: Experience of a Single Tertiary Center

**DOI:** 10.21470/1678-9741-2019-0055

**Published:** 2020

**Authors:** Sertac Haydin, Serhat Bahadır Genç, Erkut Ozturk, Okan Yıldız, Mustafa Gunes, Ibrahim Cansaran Tanidir, Alper Guzeltas

**Affiliations:** 1 Department of Cardiovascular Surgery, Istanbul Saglik Bilimleri University, Mehmet Akif Ersoy Thoracic and Cardiovascular Surgery Education and Research Hospital, Istanbul, Turkey.; 2 Department of Pediatric Cardiology, Istanbul Saglik Bilimleri University, Mehmet Akif Ersoy Thoracic and Cardiovascular Surgery Education and Research Hospital, Istanbul, Turkey.

**Keywords:** Pulmonary Atresia, Heart Septal Defects, Ventricular, Constriction, Pathologic, Heart Defects Congenital, Operative Time, Respiratory System Abnormalities

## Abstract

**Objective:**

To evaluate surgical management and results of patients with pulmonary atresia and ventricular septal defect with major aortopulmonary collateral arteries (PA/VSD/MAPCAs).

**Methods:**

We reviewed a consecutive series of patients with PA/VSD/MAPCAs between January 2012 and October 2018. Study patients were separated into Group A, efficient MAPCAs; Group B, hypoplastic MAPCAs; Group C, severe hypoplastic MAPCAs at all divisions; and Group D, distal stenosis at most MAPCAs divisions.

**Results:**

Thirty-six patients were included in the study. Median age at operation time was 5.5 months (2-110 months), median weight was 8 kg (2.5-21 kg), and median number of MAPCAs was three (1-6). In Group A, 14 patients underwent single-stage total correction (TC); in Group B, 18 patients underwent unifocalization and central shunting; and in Group C, four patients had aortopulmonary window creation and collateral ligation. No patient was placed in Group D. Seventy percent of patients (n=25) had the TC operation. Early mortality was not seen in Group A, but the other two groups had a 13.6% mortality rate. At the follow-up, three patients had reintervention, two had new conduit replacement, and one had right ventricular outflow tract reconstruction.

**Conclusion:**

Evaluating patients with PA/VSD/MAPCAs in detail and subdividing them is quite useful in determining the appropriate surgical approach. With this strategy, TC can be achieved in most patients. Single-stage TC is better than other surgical methods due to its lower mortality and reintervention rates. Care should be taken in terms of early postoperative intensive care complications and reintervention indications during follow-ups.

**Table:** 

Abbreviations, acronyms & symbols				
AP	= Aortopulmonary		Pa	= Pulmonary artery
APW-cL	= Aortopulmonary window creation and collateral ligation		PA	= Pulmonary atresia
CO	= Cardiac output		PAP	= Pulmonary arterial pressure
cPa	= Central pulmonary artery		Pas	= Pulmonary arteries
CPB	= Cardiopulmonary bypass		RV	= Right ventricle
CS-UF	= Central shunting after unifocalization		RVSP	= Right ventricular systolic pressure
CT	= Computed tomography		SBP	= Systolic blood pressure
ECMO	= Extracorporeal membrane oxygenation		SPSS	= Statistical Package for the Social Sciences
ICU	= Intensive care unit		SSTC	= Single-stage total correcion
LV	= Left ventricle		SVC	= Superior vena cava
MAPCAs	= Major aortopulmonary collateral arteries		TC	= Total correction
MV	= Mechanical ventilation		VSD	= Ventricular septal defect

## INTRODUCTION

Pulmonary atresia with ventricular septal defect (PA/VSD) is a relatively rare, complex, and heterogeneous form of congenital heart disease^[1]^. The survival rate of this disease without intervention is low, with the 10-year and 20-year survival rates reported at 50% and 10%, respectively, in the literature^[2]^. Treatment and clinical practice differ among subgroups of PA/VSD; however, the most important of these groups is the PA/VSD with major aortopulmonary collateral arteries (MAPCAs).

It is evident that the optimal surgical treatment of PA/VSD/MAPCAs remains controversial. Different strategies are being performed, such as providing right ventricle (RV) to pulmonary artery (Pa) continuity by single or multistage operations, with or without intracardiac repair; central shunting with unifocalization; and creation of an aortopulmonary (AP) window according to the adequacy of the length of MAPCAs, presence of stenotic segments and native pulmonary arteries (Pas), and status of distal pulmonary vascular bed^[3-7]^.

In this subgroup, the main objective is to repair the interventricular connection (VSD repair) to achieve a biventricular, separate circulation and to maintain an unobstructed RV to Pa connection. It is very important and fundamental to reconstruct the pulmonary arterial tree through adequate MAPCAs unifocalization and patch repairs without any obstruction in order to maintain a low-resistance pulmonary vascular bed with a low right ventricular systolic pressure (RVSP)^[3,4]^.

This paper presents the early and midterm surgical strategies and results of PA/VSD/MAPCAs patients.

## METHODS

We reviewed a consecutive series of patients with PA/VSD/MAPCAs between January 2012 and October 2018. Patients with PA/VSD with no MAPCAs, patients who had been operated on at any stage by another clinic, and single-ventricle patients were excluded from the study. This retrospective study was approved by the institutional ethics committee and was conducted in accordance with the principles of the Declaration of Helsinki.

The preoperative demographic data (gender, weight, and additional genetic syndromes), previous history, preoperative and postoperative echocardiography reports, surgical data, additional cardiac defects, clinical follow-up, and postoperative intensive care unit (ICU) reports of the study group were evaluated retrospectively.

Cardiac catheterization and angiography were performed in all patients as soon as possible following the diagnosis. Additionally, 128-slice dual-source computed tomography (CT) angiography was performed in all patients after its establishment in 2015.

The origin, number, magnitude, and parenchymal distribution of the MAPCAs, the pulmonary segmental regions that were supplied by MAPCAs, and the presence of native Pas and their magnitude were evaluated in detail. Single-supply MAPCAs and dual-supply MAPCAs (defined by > 15 lung segments with dual supply from both the native Pa system and the MAPCAs) were interpreted. These evaluations were also supported by surgical data (anatomic and intraoperative pulmonary flow studies).

Patients were divided into four major Groups (A, B, C, and D), based on the detailed evaluations mentioned above^[5,7-9]^.

In Group A, MAPCAs were sufficient in size and well distributed to the distal lung segments. Complete unifocalization enabled the VSD closure and RV-Pa connection (with a conduit or transannular patch repair) after an intraoperative pulmonary flow study (mean pulmonary arterial pressure [PAP] < 25 mmHg)^[5]^.

In Group B, there were hypoplastic MAPCAs or MAPCAs with stenotic segments. Patients with intraoperative flow study (mean PAP > 25 mmHg) in Group A were also evaluated under Group B. This group of patients was treated without an intracardiac repair. They underwent unifocalization with a central shunt operation.

In Group C, confluent native hypoplastic Pas arborize to all lung segments, such that all MAPCAs were dual supply. Flow study was not performed in this group. AP window was the choice of surgery with ligations of Pa-related collaterals.

In Group D, there were MAPCAs with distal segmental stenosis. The surgical strategy in this group of patients was planned to be staged unifocalization of MAPCAs with lateral thoracotomy.

A total number of 36 patients were divided into Group A (n=14), Group B (n=18), and Group C (n=4). None of the patients was placed in Group D.

### Surgical Strategy and Technique

Unifocalization of MAPCAs in Groups A and B was performed through a median sternotomy, as described by the Stanford group^[5]^. If there was a central pulmonary artery (cPa), the cPa and its branches were initially mobilized and dissected. The posterior mediastinum was dissected through a window by retracting the ascending aorta to the left, the superior vena cava (SVC) to the right, and the right bronchus to the cranial side. If possible, each MAPCA was identified before cardiopulmonary bypass (CPB) or heparin infusion. On CPB, MAPCAs were first encircled with vessel loops or occluded by a Yasargil neurovascular clip. By pulling on collaterals, their central dissection was performed, and their origin was definitively occluded using double hemostatic surgical clips. At this stage, thorough dissection of the collaterals and their ramifications was performed. The esophagus should be identified and protected during the dissection. We emphasize that special attention must be paid to the hilar distribution of each collateral with respect to the ipsilateral bronchial system. Ideally, the arterial branching of the pulmonary vessels should be in front of the airways in both lungs: this was the goal of unifocalization. All dissected and/or translocated collaterals were then distally clipped with Yasargil neurovascular clips (Aesculap AG & Co., Tuttlingen, Germany) and placed in a direction parallel to their distal branching to avoid distortion. The unifocalization procedure was then performed on full CPB with a beating empty heart. Dissected and translocated collaterals always provided enough tissue for direct anastomosis. All anastomoses (end-to-end for confluence reconstruction, side-to-side in all other cases) were performed with 8-0 running polypropylene sutures, and they were made as long as possible to avoid stenosis. Of course, native Pas, whenever present, were included by opening them longitudinally and using them as the basis for unifocalization. After unifocalization was completed, the cPas were enlarged with fresh autologous pericardium or with heterologous pericardium from hilum to hilum after pulmonary plasty was completed.

The left atrial vent was advanced into the left ventricle (LV) for the flow study. The lungs were ventilated, and the heart was beating. A line derived from the arterial port of the oxygenator and controlled by a roller pump was debubbled, introduced, and snagged into the pericardium together with a line for pressure recording. Using a 25% stepwise increase, a maximum blood flow of 3 L/min per m^2^ of body surface area was pumped into the reconstructed pulmonary arterial circulation, and the mean PAP was simultaneously recorded. The mean PAP oscillation was recorded for each incremental step of the flow study.

At full flow, with mean PAP < 25 mmHg, patients proceeded to simultaneous VSD repair. At this stage, all VSD repairs were performed after aortic cross-clamping and cardiac protection with del Nido cardioplegia solution. A glutaraldehyde-treated autologous pericardial patch, or a bovine pericardial patch when autologous pericardium was unavailable, was used for VSD closure. A valved conduit (homograft, Contegra, etc.) was placed to connect the RV with the reconstructed neopulmonary arterial system. Single-stage complete correction referred to the complete unifocalization and repair performed during a single procedure without any prior interventions.

If mean PAP > 25 mmHg, a central shunt was created with a polytetrafluoroethylene graft. Fenestrated VSD closure was not used in any patient. A longitudinal incision was made on the reconstructed neopulmonary artery. A Gore-Tex conduit (5 or 6 mm) (W.L. Gore & Assoc, Flagstaff, Arizona, USA) was anastomosed end-to-side to the main Pa, and the distal end of the conduit was cut transversely as described by the Melbourne group^[7]^.

If the patient was in Group C (native Pas were confluent and arborized to all lung segments, *i.e*., all MAPCAs were dual supply), regardless of the systemic oxygen saturation, a surgical AP window was created as a central shunt to promote the growth of the native Pa system.

### Statistical Analysis

The Statistical Package for the Social Sciences (SPSS) software for Windows (SPSS, Chicago, Illinois, USA), version 15, was used for statistical analysis. Continuous variables were expressed as median (minimum–maximum) or mean ± standard deviation; categorical variables were expressed as percentages. Comparison of actuarial curves was performed using the Cox regression analysis.

## RESULTS

### Patients

A total of 36 patients (21 males and 15 females) had PA/VSD with MAPCAs. Seven patients (19%) had DiGeorge syndrome. The median age at the time of operation was 5.5 months (range 0.16-9.1 years). The median weight at the time of surgery was 8 kg (range 3.5-21 kg).

Each patient had a catheter angiography, and 21 of them had a CT angiography. Single-supply MAPCAs and dual-supply MAPCAs were detected prior to the surgery (n=32 and n=4, respectively).

The median number of MAPCAs was three (range 1-6). This included a median of three MAPCAs to the right lung (range 1-5) and a median of two MAPCAs to the left lung (range 1-6). Right-sided arcus aorta was seen in four patients. Preoperatively, 11 patients had clinical heart failure not amenable to medical management, and therefore underwent early surgical treatment; five patients were severely cyanotic (70%). Twenty patients were asymptomatic. The patients’ demographic characteristics are listed in Table 1.

**Table 1:** Demonstration of patients' characteristics.

Characteristic	n=36
Sex (male/female)	21/15
Genetic syndrome (yes/no)	7/29
Age (months)	5.5 (2-110)
< 6 months	16 (44%)
6-12 months	9 (25%)
> 12 months	11 (31%)
Body weight (kg)	8 (3.5-21)
Echocardiographic variables	
LV short fraction (%)	36 (24-44)
LV ejection fraction (%)	65 (50-75)
Associated anomaly	
Atrial septal defect	5 (14%)
Patent foramen ovale	20 (56%)
Right arcus aorta	4 (11%)
Catheter angiography and/or CT angiography	
MAPCAs (single/dual)	32/4
Right lung	3 (1-5)
Left lung	2 (1-6)
Native pulmonary arteries (yes/no)	29/7
Group	
A	14
B	18
C	4
D	-

CT=computed tomography; LV=left ventricle; MAPCAs=major aortopulmonary collateral arteries Values are median (range) or n (%)

### Surgery

All patients in Group A (n=14) had single-stage total correction (SSTC), Group B (n=18) underwent central shunting after unifocalization (CS-UF), and Group C (n=4) underwent aortopulmonary window creation with collateral ligation (APW-cL). Median CPB time was 70 minutes (range 100-370 min).

Pa reconstruction was performed with fresh autologous pericardium (n=9) with the CorMatrix tube (CorMatrix, Atlanta, Georgia, USA) (n=5) or Matrix P plus N (AutoTissue, Berlin, Germany) (n=3). The RV-Pa connection was maintained with bovine jugular conduit (Contegra, Medtronic, Inc., Minneapolis, Minnesota, USA) in 13 patients, a Matrix P® conduit (AutoTissue, Berlin, Germany) in seven patients, and a Labcor graft (Sulzer Carbomedics, Austin, Texas, USA) in four patients. The other two patients had a homograft (n=1) and a Hancock conduit (Medtronic, Minneapolis, Minnesota, USA) (n=1). Median conduit size was 17 mm (range 13-19mm).

**Fig. 1:**
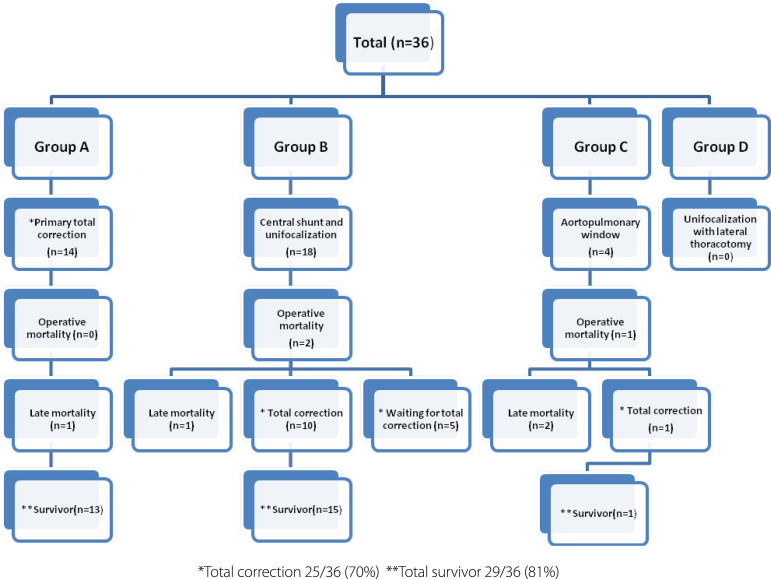
Flow diagram for the 36 patients with pulmonary atresia and ventricular septal defect with major aortopulmonary collateral arteries. *Total correction 25/36 (70%) **Total survivor 29/36 (81%)

VSD was repaired with a glutaraldehyde-treated autologous pericardial patch in 22 patients and with a bovine pericardial patch in four patients. Central shunt sizes were 3.5 mm (n=2), 4 mm (n=2), 5 mm (n=7), and 6 mm (n=7). Ten out of 18 patients who had CS-UF and one out of four patients in the APW-cL group underwent total correction followed by intracardiac repair. Total complete repair was then performed in 70% (25/36) of the patients. A flowchart demonstrating the surgical approaches is shown in Figure 1.

Pressures and pressure ratios measured in the operating room after CPB were evaluated, the RVSP/systolic blood pressure (SBP) ratio was < 50% for 80% of the total correction patients. However, two patients had RVSP/SBP ratio > 65%.

### Intensive Care Unit

The sternum was left open in 12 cases (33%). Mean duration of mechanical ventilation (MV) was 4 ± 3.1 days. Duration of ICU stay was 8 ± 5 days, and postoperative hospitalization was 21 ± 12 days.

Six patients had antiarrhythmic drug therapy for hemodynamically significant arrhythmias (junctional ectopic tachycardia [n=3], ventricular tachycardia [n=1], focal atrial tachycardia [n=1]). Five patients had peritoneal dialysis because of low cardiac output (CO). Nitric oxide inhalation therapy was used in six patients for high right ventricular pressure.

Postoperative infection occurred in six patients. Three of these six patients had a Gram-negative bacterial septicemia with positive blood cultures, and one had a urinary tract infection. The other two patients had ventilator-associated pneumonia.

Transient phrenic nerve paresis was detected in one patient and chylothorax in another. A third patient had pleural effusion.

Pulmonary haemorrhagia occurred in three patients, and one patient had gastric bleeding.

Multiple reintubations due to bronchial hyperreactivity and pulmonary edema were needed in 70% of the patients (95% within two years of the first operation).

Two of the patients in the CS-UF group needed extracorporeal membrane oxygenation (ECMO) support; these patients were weaned successfully.

Angiography was performed in two patients at the early postoperative period. Total correction was performed in one of these patients and he/she was reoperated due to conduit stenosis. Other patient was the one in the CS-UF under ECMO support group. His shunt was revised due to shunt obstruction.

There were three in-hospital deaths (8.3%). Two had CS-UF surgery, and the other had APW-cL. Two patients died within the first 48 hours. Low CO developed in one of them during the first 24 hours postoperatively. Extracorporeal life support was not attempted for this patient since the baby has syndromic appearance and associated comorbidities. The other patient had a sudden cardiac arrest of an unknown etiology and died on the 16^th^ postoperative day with *Klebsiella pneumoniae*-related septic shock.

### Follow-Up

Median follow-up time was three years (range 2 months-6 years). Four patients died during the interstage period. The actuarial survival curve for the 14 patients who underwent a SSTC and for the 18 patients who underwent a unifocalization and central shunt shows that the three-year survival was 83% *vs*. 77%, respectively (*P*=0.04).

After total correction, three patients needed reintervention, and two of these had RV-Pa conduit exchange. Another patient had an operation for RV outlet reconstruction. Six patients in the CS-UF group had reinterventions. The three-year survival free-from-reintervention rate was 77% in Group A and 67% in Group B (*P*=0.03).

## DISCUSSION

In our study, we evaluated the results of surgical approaches in patients with PA/VSD/MAPCAs at a tertiary center. After initial diagnosis in these complex and heterogeneous patients, it was found that dividing them into subgroups was useful after performing a detailed evaluation with echocardiography, CT angiography, and catheter angiography. SSTC as an initial plan was the most appropriate surgical choice, with acceptable mortality rates in suitable patients. Because of the high mortality and morbidity rates during interstage phases, close clinical follow-up in these patients is crucial.

Surgical management of PA with MAPCAs has improved the prognosis for this complex heart disease, but the timing and surgical options are still in evolution. Optimal timing for surgical intervention for PA/VSD/MAPCA should be between three and six months^[10,11]^. In some circumstances, however, intervention cannot postpone until the ideal three to six months period. An earlier approach is necessary in three circumstances: 1) cardiopulmonary instability (profound cyanosis or overcirculation), 2) the presence of a unilateral ductus arteriosus providing flow to one lung, and 3) the presence of centrally confluent but very hypoplastic true Pas that completely arborize to all lung segments and are supplied by multiple “dual supply” collaterals. In the Stanford group, the median age at the time of operation was 4.5 months, with a range of 0.1-11.5 years^[5]^. The median operation age in our study was 5.5 months (2-110 months), which is in close range with the Stanford group.

Due to the improvement of surgical techniques and equipment, surgical procedures can be performed more easily, with high success and low mortality rates, and earlier than it was used to be. During surgery, it is crucial to connect as much MAPCAs as possible; therefore, aiming to achieve early unifocalization is gaining importance among more clinics. There are two major approaches to surgical technique, which are defined by the Melbourne and Stanford groups^[3,4,8]^.

The Melbourne group^[7]^ performed the pulmonary rehabilitation process with a completely natural Pa system-based strategy. They asserted that unifocalization and/or embolization of MAPCAs were not necessary. This was based on their findings that in a series of 60 unifocalized MAPCAs, 26 vessels had thrombosed and 12 had narrowed more than > 50% at a mean of 3.4 years. This strategy resulted in complete repair in 73% of patients. The mortality rate due to noncardiac causes was 10%. In other published literature, Chen et al.^[8]^ had a complete rehabilitation strategy and their complete repair rate was 60%; and the RV/LV pressure ratio was > 0.50 in more than half the patients. Likewise, Dragelusco et al.^[9]^ reported a mortality rate of 15% and a complete repair rate of 70% in a series of 20 patients.

In contrast, the Stanford group^[10]^ proposed a new algorithmic approach to the diagnostic workup and treatment of PA/VSD/MAPCAs in 2009. They emphasized that catheterization should be a part of surgical planning and carried out at the initial diagnosis time. It should indicate all details of pulmonary blood flow (true Pa branching, outflow distribution of MAPCAs, all connections between true Pa and MAPCA, the presence of dual supply lung segments, and stenosis status in all collaterals and distal pressure measures, if necessary). After detailed diagnostic workups, a rational surgical approach needs to be performed. The Stanford group published the world’s largest series (307 patients) in 2018. In their algorithmic approach, 93% of patients achieved total correction, and the mortality was 3.5-fold lower in patients who received SSTC than in those who did not received it. In other paths (aortopulmonary window, central shunt, and unifocalization), early mortality was determined as 4%^[5]^.

In our series, we performed an algorithmic approach similar to the one from the Stanford group^[11]^, though our total correction rate was lower than theirs (70% *vs*. 93%, respectively). Additionally, our mortality rate was 0% in SSTC patients but higher (13.6%) in other surgical paths. Retrospective analysis of our results and other published results, from the point of total correction rate, revealed that this situation could be due to some patients with unifocalization with central shunting not being totally corrected yet. Also, we realized that in some patients, although they were suitable for SSTC, other types of surgical palliations were performed due to our inexperience, and exceedingly more losses occurred during the interstage period.

An intraoperative flow study constitutes an important stage in making the primary total correction decision, and therefore the VSD closure decision. Following unifocalization completion, while still on CPB, PAP is continuously measured while pulmonary blood flow through a separate perfusion circuit is increased incrementally to a full CO (3 L/min/m^2^). If the mean PAP remains < 25 mmHg at a full flow, an RV/LV pressure ratio < 0.5 can be expected following VSD closure^[11]^. In a study of 20 patients reported from Toronto (2009), it was shown that the intraoperative flow study was more effective in making total correction when compared to the preoperative study of anatomy. It was also stated that the VSD could be closed with a flow of 2.5 L/min/m^2^ and mean PAP < 30 mmHg^[12]^. In our center, after surgical unifocalization of the Pas, we closed the VSD and placed a RV-PA conduit if the mean PAP remained < 25 mmHg at a full flow. If the mean PAP was > 25 mmHg, those patients underwent central shunt operation.

Different complications may occur during the early postoperative period, depending on the type of surgical procedure; this may cause mortality or morbidity. Complications directly related to the unifocalization procedure include phrenic nerve palsy, recurrent nerve palsy, pulmonary hemorrhage, gastric paralysis, and MAPCAs stump aneurysm^[14,15]^. In our study, similar complications were reported but they did not lead to mortality.

Some important factors causing postoperative complications in PA/VSD/MAPCAs are persistent airway hypersensitivity and respiratory problems^[16,17]^. For example, Asija et al.^[18]^ reported reperfusion edema in 50% of patients in their studies, and Ackerman et al.^[19]^ diagnosed airway hypersensitivity with methacholine in 22 out of 33 patients in their study series (66%, eight of 13 syndromic patients and 14 of 20 non-syndromic patients). In our study, recurrent intubations due to bronchial hyperreactivity were observed in 70% of the patients (95% initial patients). The duration of MV time was prolonged in these patients. Recently, we observed that aggressive inhaler bronchodilator and steroid treatment in these patients and the use of high-flow oxygen treatment caused a gradual decrease in this rate.

Reintervention due to unifocalized MAPCA stenosis is another important problem during these patients’ follow-ups. Pulsatile stress, hypoxia, and hyperoxia seem to disrupt the elastic lamina and smooth muscle cell migration, which eventually leads to obstruction. As the understanding of MAPCAs’ pathophysiologic behavior increases, we may overcome long-term complications. Carotti et al.^[13]^ reported a 52% freedom-from-percutaneous intervention rate after 14 years of unifocalization. Freedom from any Pa reintervention was 64% at five years in the Stanford group^[20]^. Pa reinterventions are crucial to maintain the right ventricular pressure as low as possible. A combination of catheter and surgical interventions is an effective treatment for stenosis/narrowing Pas. In our study, the reintervention rates in Groups A and B were 21% and 35%, respectively; these low reintervention rates were primarily due to the shorter follow-up period than the above-referenced studies.

### Limitations

This nature of a single-center, retrospective study is the major limitation. Variations in the preoperative angiography quality limited the certainty of absolute counts for the minimum number of AP collaterals needed to be unifocalized, as well as the number of distal stenoses. Some patients were not preoperatively evaluated with CT angiography, and the initial patient outcomes might have been affected by the learning curve and an unsettled clinical algorithm.

## CONCLUSION

The management of patients with PA/VSD/MAPCAs is unique and complicated. The preoperative clinical status, imaging methods (echocardiography, catheterization and angiography, and CT angiography), and operative findings might be helpful for planning the most suitable management. Classifying patients based on the MAPCA properties and presence of Pas might help doctors to determine the best surgical strategy to use. Although SSTC with low mortality and reintervention rates seems to be the ideal surgical strategy, other staged surgical strategies enable total correction in many patients for whom single-stage correction is not possible. Doctors should be careful about ICU complications and residual and recurrent problems at follow-ups.

**Table:** 

Author's roles & responsibilities	
SH	Substantial contributions to the conception or design of the work; agreement to be accountable for all aspects of the work in ensuring that questions related to the accuracy or integrity of any part of the work are appropriately investigated and resolved; final approval of the version to be published
SBG	Drafting the work or revising it critically for important intellectual content; agreement to be accountable for all aspects of the work in ensuring that questions related to the accuracy or integrity of any part of the work are appropriately investigated and resolved; final approval of the version to be published
EO	Substantial contributions to the conception or design of the work; drafting the work or revising it critically for important intellectual content; agreement to be accountable for all aspects of the work in ensuring that questions related to the accuracy or integrity of any part of the work are appropriately investigated and resolved; final approval of the version to be published
OY	The acquisition, analysis, or interpretation of data for the work; agreement to be accountable for all aspects of the work in ensuring that questions related to the accuracy or integrity of any part of the work are appropriately investigated and resolved; final approval of the version to be published
MG	The acquisition, analysis, or interpretation of data for the work; drafting the work or revising it critically for important intellectual content; agreement to be accountable for all aspects of the work in ensuring that questions related to the accuracy or integrity of any part of the work are appropriately investigated and resolved; final approval of the version to be published
ICT	The acquisition, analysis, or interpretation of data for the work; agreement to be accountable for all aspects of the work in ensuring that questions related to the accuracy or integrity of any part of the work are appropriately investigated and resolved; final approval of the version to be published
AG	Substantial contributions to the conception or design of the work; drafting the work or revising it critically for important intellectual content; agreement to be accountable for all aspects of the work in ensuring that questions related to the accuracy or integrity of any part of the work are appropriately investigated and resolved; final approval of the version to be published
